# Clinical implementation of rapid *CYP2C19* genotyping to guide antiplatelet therapy after percutaneous coronary intervention

**DOI:** 10.1186/s12967-018-1469-8

**Published:** 2018-04-11

**Authors:** Larisa H. Cavallari, Francesco Franchi, Fabiana Rollini, Latonya Been, Andrea Rivas, Malhar Agarwal, D. Max Smith, Kimberly Newsom, Yan Gong, Amanda R. Elsey, Petr Starostik, Julie A. Johnson, Dominick J. Angiolillo

**Affiliations:** 1 Department of Pharmacotherapy and Translational Research, University of Florida, Gainesville, FL USA; 2 Center for Pharmacogenomics, University of Florida, Gainesville, FL USA; 3 Clinical and Translational Science Institute, University of Florida, Gainesville, FL USA; 4 Division of Cardiology, Department of Medicine, University of Florida, Jacksonville, FL USA; 5 University of Florida Health Pathology Laboratories, Gainesville, FL USA; 6 Department of Pathology, Immunology and Laboratory Medicine, University of Florida, Gainesville, FL USA

**Keywords:** CYP2C19, Genotype, Clopidogrel, Percutaneous coronary intervention, Ticagrelor, Prasugrel

## Abstract

**Background:**

The *CYP2C19* nonfunctional genotype reduces clopidogrel effectiveness after percutaneous coronary intervention (PCI). Following clinical implementation of *CYP2C19* genotyping at University Florida (UF) Health Shands Hospital in 2012, where genotype results are available approximately 3 days after PCI, testing was expanded to UF Health Jacksonville in 2016 utilizing a rapid genotyping approach. We describe metrics with this latter implementation.

**Methods:**

Patients at UF Health Jacksonville undergoing left heart catheterization with intent to undergo PCI were targeted for genotyping using the Spartan RX™ system. Testing metrics and provider acceptance of testing and response to genotype results were examined, as was antiplatelet therapy over the 6 months following genotyping.

**Results:**

In the first year, 931 patients, including 392/505 (78%) total patients undergoing PCI, were genotyped. The median genotype test turnaround time was 96 min. Genotype results were available for 388 (99%) PCI patients prior to discharge. Of 336 genotyped PCI patients alive at discharge and not enrolled in an antiplatelet therapy trial, 1/6 (17%) poor metabolizers (PMs, with two nonfunctional alleles), 38/93 (41%) intermediate metabolizers (IMs, with one nonfunctional allele), and 119/237 (50%) patients without a nonfunctional allele were prescribed clopidogrel (p = 0.110). Clopidogrel use was higher among non-ACS versus ACS patients (78.6% vs. 42.2%, p < 0.001). Six months later, among patients with follow-up data, clopidogrel was prescribed in 0/4 (0%) PMs, 33/65 (51%) IMs, and 115/182 (63%) patients without a nonfunctional allele (p = 0.008 across groups; p = 0.020 for PMs versus those without a nonfunctional allele).

**Conclusion:**

These data demonstrate that rapid genotyping is clinically feasible at a high volume cardiac catheterization facility and allows informed chronic antiplatelet prescribing, with lower clopidogrel use in PMs at 6 months.

*Trial registration* ClinicalTrials.gov Identifier: NCT02724319; registered March 31, 2016; https://www.clinicaltrials.gov/ct2/show/NCT02724319?term=angiolillo&rank=7

## Background

Dual antiplatelet therapy with aspirin and a P2Y_12_ receptor inhibitor is the standard of care for patients with coronary artery disease (CAD) undergoing percutaneous coronary intervention (PCI) [1, 2]. Currently, three oral P2Y_12_ receptor antagonists (clopidogrel, prasugrel, and ticagrelor) are clinically available. Clopidogrel remains broadly utilized, which may be attributed to its lower cost, given that it is available in a generic formulation, and its expanded indications compared with prasugrel and ticagrelor [3, 4]. Prasugrel and ticagrelor in fact have an indication only for the treatment of patients with acute coronary syndrome (ACS), while clopidogrel is the only oral P2Y_12_ receptor antagonist also with an indication for the treatment of stable CAD [5]. Compared to clopidogrel, prasugrel and ticagrelor lead to a greater reduction in atherothrombotic events, albeit at the expense of increased bleeding not related to coronary artery bypass grafting, but clopidogrel is still frequently used [3, 4, 6].

Clopidogrel is a thienopyridine that requires bioactivation to an active metabolite that irreversibly binds the platelet P2Y_12_ receptor and inhibits platelet activation and subsequent aggregation [3, 5]. There is significant inter-patient variability in clopidogrel-induced antiplatelet effects, which has important prognostic implications [7, 8]. In particular, in patients undergoing PCI, studies have consistently shown that those with reduced clopidogrel-induced antiplatelet effects, who thus persist with high on-clopidogrel platelet reactivity, are at high risk for ischemic recurrences, including stent thrombosis [8–11]. Emerging studies have also shown that the presence of enhanced clopidogrel-induced antiplatelet effects, leading to low platelet reactivity, may increase the risk of bleeding complications [9]. Multiple factors, including clinical, cellular, and genetic factors, may contribute to interindividual response variability to clopidogrel [7]. Among the genetic factors, polymorphisms in the *CYP2C19* gene have consistently shown to have a role [12–14].

The CYP2C19 enzyme is involved in both metabolic steps mediating the biotransformation of clopidogrel to its pharmacologically active form. The CYP2C19*2 and *3 alleles are referred to as nonfunctional alleles and confer absent enzyme activity. These alleles are associated with lower plasma concentrations of the active metabolite and reduced platelet inhibition with clopidogrel [15]. Studies conducted in clopidogrel-treated patients undergoing PCI have shown an increased risk for ischemic events, in particular stent thrombosis, in the presence of a nonfunctional allele [12, 16]. Prasugrel and ticagrelor are newer P2Y_12_ receptor inhibitors that are not affected by the *CYP2C19* genotype [17, 18], and consortium guidelines recommend consideration of these agents in patients with a nonfunctional genotype [14].

In June 2012, *CYP2C19* testing on a GenMark Dx^®^ platform (GenMark Diagnostics, Inc., Carlsbad, CA) was launched at the University of Florida (UF) Health Shands Hospital in Gainesville to assist with selection of antiplatelet therapy after PCI, and metrics with this implementation have been described [19]. This was a pharmacist-led effort. In April 2016, in a physician-led effort, *CYP2C19* testing was launched at UF Health Jacksonville, whereby patients undergoing left heart catheterization with intent to undergo PCI were genotyped on a Spartan RX™ platform (Spartan Bioscience Inc, Ottawa, ON). This provided experience with a rapid genotyping approach to clinical *CYP2C19* testing. Herein we describe implementation metrics associated with *CYP2C19* genotype-guided antiplatelet therapy at UF Health Jacksonville. We specifically report on physician uptake of *CYP2C19* testing, genotype results and turnaround time, and both acute and chronic antiplatelet therapy prescribed after genotype results were available. We also describe lessons learned based on our experiences with two approaches to *CYP2C19* genotyping implementation.

## Methods

### Procedures for implementation

Patients undergoing emergent or planned left heart catheterization with intent to undergo PCI were targeted for testing. Patients with a history of human immunodeficiency virus or hepatitis C virus were excluded. Written informed consent for clinical *CYP2C19* testing and collection of clinical data and a blood sample for future research were obtained from each patient. Genetic samples were collected via a buccal swab prior to cardiac catheterization when possible. For patients undergoing emergency procedures (e.g., ST-segment elevation myocardial infarction) or otherwise unable to provide consent prior to catheterization (e.g., patients with cardiogenic shock or intubated), informed consent and genetic sampling were obtained following PCI after patients were stabilized. Patients not receiving PCI were not tested after diagnostic left heart catheterization.

Genetic samples were transported to the college of American pathologist/CLIA licensed pathology laboratory, located five floors below the cardiac catheterization laboratory. Samples were processed using the Spartan RX™ system, which tests for the *CYP2C19* nonfunctional *2 (c.681G>A; rs4244285) and *3 (c.636G>A; rs4986893) alleles and the increased function *17 (c.-806C>T; rs12248560) allele [20]. Those lacking a *2, *3, or *17 allele were assigned the *1 allele designation. Phenotypes were assigned according to standardized nomenclature, with PMs having two nonfunctional alleles (e.g. *2/*2), IMs having a single nonfunctional allele (e.g. *1/*2, *2/*17), normal metabolizers (NMs) having the *1/*1 genotype, and rapid and ultra-rapid metabolizers (RMs and UMs) having the *1/*17 and *17/*17 genotypes, respectively [21]. One Spartan RX™ system was initially available, but a second system was added in May 2017 to accommodate testing multiple patients at once. In the event of inconclusive genotype results, collection of an additional buccal cell sample for genotyping was attempted. Testing was only available Monday through Friday. For patients presenting on the weekend for emergency PCI, samples were collected on Monday for genotyping if the patient remained hospitalized.

The overall goal was to have genetic information available by the end of the PCI procedure or before discharge for patients unable to provide informed consent prior to coronary intervention. Genotype and phenotype results were placed in the Epic electronic health record (EHR) under the laboratory reports tab, with phenotype assignment as described above. Results were communicated to physicians via an inbox message to use at their discretion taking into consideration other factors (e.g., clinical presentation, cardiovascular risk factors, concomitant medications, insurance, PCI complexity) in making antiplatelet therapy decisions. No clinical pharmacist support was provided. Genotyping kits and systems were donated by Spartan Biosciences, Inc. Technical and pathologist time was paid through grant support.

### Data collection and analysis

Data for the first 12 months of implementation were collected on the genotype test ordering rate, genotype turnaround time (defined as the time between the sample arrival in the pathology laboratory to the time the report was signed by the pathologist) and genotype results. For patients receiving PCI, data were also collected on antiplatelet therapy at discharge and 6 months after coronary intervention. Metrics data were tracked through review of patient encounters in the EHR and telephone calls. Use of clopidogrel was compared between PMs, IMs, and patients without a nonfunctional allele (e.g., combination of NMs, RMs, and UMs) using Chi square analysis or the Fisher’s exact test. Changes in antiplatelet therapy from baseline to 6 months were assessed within each phenotype group using the McNemar test. All analyses were conducted using SAS version 9.4 (SAS, Cary, NC). Study procedures and data collection were approved by the Institutional Review Board at UF Health Jacksonville, and all procedures were in accordance with the ethical standards of the Declaration of Helsinki.

## Results

From April 28, 2016 to April 27, 2017, 931 patients undergoing left heart catheterization for suspected coronary disease were genotyped, representing 68% (931/1366) of all patients undergoing left heart catheterization during that period (Table 1). The median genotype test turnaround time was 96 (interquartile range of 78–144) min. Genotyping for 129 patients (14%) was unsuccessful with the initial sample (56 inconclusive results, 73 device errors). One additional sample was collected in 113 patients, two additional samples were collected in 10 patients, and 6 patients refused sample recollection. Nine of the 123 patients with additional sample collection had multiple inconclusive results.

**Table 1:** *CYP2C19* test ordering and adoption rates in the first 12 months at UF Health Jacksonville

Implementation metric	No. of patients (%)
Total number of patients who underwent left heart catheterization	1366
Patients who were genotyped	931
Genotype test adoption rate	931/1366 (68%)
Genotypes successfully completed with initial sample	802 (86%)
Total number of patients who underwent PCI	505
PCI patients who were genotyped	392
Genotype test adoption rate	392/505 (78%)
Patients included in the analysis of antiplatelet therapy at discharge	336
Patients included in the analysis of antiplatelet therapy at 6 months	258

*PCI* percutaneous coronary intervention

A total of 392 of the 931 genotyped patients (42%) underwent PCI, representing 78% (392/505) of all patients undergoing PCI during the 12 month period. Of these, 4 (1%) had inconclusive genotype results. Of the 113 patients receiving PCI and not genotyped, 12 refused to participate in the study and the remaining 101 were excluded, unable to provide informed consent, or not approached about testing (e.g. underwent PCI over the weekend and were discharged before they could be consented). The characteristics of the genotyped patients receiving PCI are shown in Table 2. The majority were male and presented with an ACS at the time of PCI. Twenty-nine percent had a *2 allele; 27% were intermediate metabolizers (IMs) and 2% were poor metabolizers (PMs). The *3 allele was not detected in any patient.

**Table 2:** Characteristics of patients who underwent PCI and *CYP2C19* genotyping over the first 12 months

Characteristic	n = 392
Age (years)	63 ± 11
Male sex	271 (69)
Race	
White	292 (74.5)
Black	93 (23.7)
Asian	3 (0.8)
Other or not reported	4 (1.0)
Past medical history	
Stroke or TIA	48 (12.2)
Gastrointestinal hemorrhage	7 (1.8)
Intracranial hemorrhage	2 (0.5)
PCI indication	
STEMI	74 (18.9)
NSTEMI	99 (25.2)
Unstable angina	174 (44.4)
Stable coronary disease	45 (11.5)
P2Y_12_ inhibitor on admission	
Clopidogrel	99 (25.3)
Prasugrel	9 (2.3)
Ticagrelor	13 (3.3)
Other or not available	11 (2.8)
Anticoagulant on admission^a^	
Warfarin	9 (2.3)
Direct oral anticoagulant	12 (3.0)
Low molecular weight heparin	1 (0.3)
Not available	9 (2.3)
Anticoagulant at discharge^a^	
Warfarin	16 (4.1)
Direct oral anticoagulant	16 (4.1)
Low molecular weight heparin	1 (0.3)
CYP2C19 phenotype	
Poor metabolizer (*2/*2)	7 (1.8)
Intermediate metabolizer (*1/*2, *2/*17)	106 (27.0)
Normal metabolizer (*1/*1)	145 (37.0)
Rapid metabolizer (*1/*17)	110 (28.1)
Ultra-rapid metabolizer (*17/*17)	20 (5.1)
Inconclusive	4 (1.0)

Mean ± SD or no. (%)

*PCI* percutaneous coronary intervention, *STEMI* ST-segment elevation myocardial infarction, *NSTEMI* non-ST-segment elevation myocardial infarction, *TIA* transient ischemic attack

^a^Warfarin, direct oral anticoagulant, or low molecular weight heparin

**Fig. 1:**
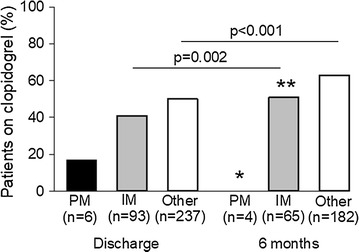
Percent of patients on clopidogrel at discharge and 6 months by CYP2C19 phenotype. *PM* poor metabolizer, *IM* intermediate metabolizer. Other includes normal metabolizers, rapid metabolizers, and ultra-rapid metabolizers. *p = 0.020 for PM versus other at 6 months. **p = 0.104 for IM versus other at 6 months

Fifty-one patients (14 with a *2 allele and 37 without a *2 allele) were enrolled in the Ticagrelor With Aspirin or Alone in High-Risk Patients After Coronary Intervention (TWILIGHT) trial (ClinicalTrials.gov Identifier: NCT02270242), which dictated treatment with ticagrelor for 3 months following PCI [22]. An additional patient died prior to hospital discharge. After excluding these 52 patients, plus 4 patients with inconclusive genotype results, 1 of 6 (17%) PMs, 38 of 93 (41%) IMs, and 119 of 237 patients without a *2 allele (50%) were placed on clopidogrel at the time of discharge (p = 0.110 for comparison across groups; Fig. 1). The remaining 178/336 patients were treated with prasugrel (81/178, 45.5%) or ticagrelor (97/178, 54.5%).

At the time of data collection for the present analysis, 6 month post-PCI follow-up data were available for 258 patients. Of 251 still taking a P2Y_12_ inhibitor, use of clopidogrel increased significantly in IMs (p = 0.002) and patients without a *2 allele (p < 0.001) from discharge to 6 months, but remained low in PMs. At 6 months, 0/4 (0%) PMs, 33/65 (51%) IMs, and 115/182 (63%) patients without a *2 allele were prescribed clopidogrel (p = 0.008 for comparison across groups). Clopidogrel use was significantly higher in those without a *2 allele compared to PMs (p = 0.020), but not compared to IMs (p = 0.104).

When stratifying antiplatelet therapy by PCI indication, there was significantly higher use of clopidogrel in non-ACS patients (n = 28) versus ACS patients (n = 223) at discharge (78.6% vs. 42.2%, p < 0.001) and 6 months (82.1% vs. 56.1%, p = 0.008). Similar to data in the whole population, among ACS patients, clopidogrel use increased significantly from baseline to 6 months in patients without a CYP2C19*2 allele (45.6% at discharge to 58.9% at 6 months, p < 0.001), as well as in IMs (35.5% at discharge to 51.6% at 6 months, p = 0.004), whereas all PMs received alternative therapy at discharge and 6 months. There were too few non-ACS patients for analysis of changes in antiplatelet therapy over time by genotype.

### Lessons learned

The implementation at UF Health Shands Hospital in Gainesville has been previously described [19]. Table 3 compares implementation approaches between UF Health Jacksonville and UF Health Shands Hospital in Gainesville and lessons learned based on experience with the two approaches. A major challenge with the genotyping approach in Gainesville was that the delay in obtaining genotype results significantly disrupted provider workflow given the need to follow up on results returned 3–4 days after the PCI procedure, which was often after the patient had been discharged. An effective communication strategy utilizing both clinical pharmacist support and electronic clinical decision support was necessary in this setting to alert providers about patients with a nonfunctional allele so that appropriate antiplatelet therapy could be instituted. This approach was successful in ensuring high uptake of prasugrel or ticagrelor in both PMs and IMs, whereas most patients without a nonfunctional allele were treated with clopidogrel. In contrast, neither pharmacist support nor clinical decision support were used to assist with antiplatelet therapy in Jacksonville where results are usually available 1–2 h after sample collection, and well before patient discharge. Also in contrast to UF Health, Gainesville, prasugrel or ticagrelor was commonly prescribed in the acute post-PCI phase at UF Health, Jacksonville regardless of genotype. Genotype was available to inform switches after the acute phase from more potent and expensive P2Y_12_ inhibitors to clopidogrel in patients without a nonfunctional allele, as evidenced by significantly increased use of clopidogrel in those without a *2 over the initial 6 month period and continued use of prasugrel or ticagrelor in PMs. Interestingly, use of clopidogrel in IMs increased over time at UF Health, Jacksonville, which is against guideline recommendations. Thus, an important lessons learned is that some sort of clinical decision support is needed to assist with genotype-guided chronic antiplatelet therapy decisions. Another lesson learned was that genotyping was useful in informing antiplatelet therapy in the setting where clopidogrel was the preferred antiplatelet agent (at UF Health, Gainesville) and in the setting where more potent P2Y_12_ inhibitors were preferred early after PCI (at UF Health, Jacksonville), with a switch to clopidogrel after the acute period, assuming that clinical decision support can be put in place to ensure appropriate genotype-guided clopidogrel use.

**Table 3:** Implementation approaches at two UF Health locations and lessons learned

Approach	UF Health Jacksonville	UF Health Gainesville	Comment
Genotyping	Rapid genotyping using a Spartan RX™ platform, with results entered into the EHR approximately 90 min from sample collection	Genotyping using a GenMark DX^®^ platform, with samples batched for processing and results entered into the EHR in an average of 3.5 days	Delays in obtaining genotype results can create significant disruptions in workflow that can be minimized when genotype results are available early after PCI Rapid genotyping is feasible for sites where the cardiac catheterization laboratory is in close proximity to the clinical pathology laboratory
Antiplatelet therapy	There was a high use of prasugrel or ticagrelor early after PCI regardless of genotype. Use of prasugrel or ticagrelor declined in the 6 months following PCI for patients without a nonfunctional allele, but remained high in PMs, with two nonfunctional alleles	There was high use of clopidogrel as the preferred antiplatelet therapy early after PCI. After genotype results were available, there was a high switch rate to prasugrel or ticagrelor in both PMs and IMs	Genotype is important to inform switches from clopidogrel to prasugrel or ticagrelor in patients with a nonfunctional allele Genotyping can also inform de-escalation from more potent P2Y_12_ inhibitors to clopidogrel in patients without a non-functional allele
Clinical pharmacy support and electronic clinical decision support	No clinical pharmacy support or electronic clinical decision support was provided	Significant pharmacist effort was devoted toward monitoring genotype test adoption and following up with physicians on test results in the first year of implementation. After the first year, pharmacist effort was limited to providing recommendations for patients with a nonfunctional allele.	Clinical pharmacist and electronic decision support is important in settings where return of genotype results is delayed Clinical pharmacist and/or electronic decision support may be necessary in settings where rapid genotyping is available to assist with decisions regarding antiplatelet therapy in the acute setting as well as decisions to de-escalation of chronic antiplatelet therapy

## Discussion

We previously showed that *CYP2C19* testing in conjunction with clinical decision support and clinical pharmacist participation can be successfully integrated into clinical care to guide antiplatelet therapy after PCI, with high prescriber adoption rates of the testing and recommendations for alternative therapy for patients with a nonfunctional allele during the initial year of the program [19]. Herein, we extend those findings based on a physician-led implementation at another UF Health institution. While there were some research aspects to the implementation, including obtaining consent for sample storage for future research, most aspects of the implementation were done as part of clinical care, with genotyping performed in a CAP/CLIA-licensed laboratory, results placed in the EHR, and prescribing decisions left to the provider. Other institutions across the United States have also clinically implemented *CYP2C1*9 testing to guide post-PCI antiplatelet therapy, with a description of strategies undertaken recently published [23]. Consistent with a recent publication from the University of Alabama, Birmingham [24], we show that, in the largest single institution study reported to date, use of a rapid *CYP2C19* genotyping platform outside of a clinical trial is clinically feasible. We further show that the availability of a rapid and user-friendly genotyping platform led to high adoption of *CYP2C19* testing for patients presenting to the cardiac catheterization laboratory with intent for PCI.

Clopidogrel reduces the risk for adverse cardiovascular events after an ACS, and additional data show reductions in the risk for cardiovascular events, including stent thrombosis, with clopidogrel use after PCI [1, 5, 6]. However, the *CYP2C19* nonfunctional genotype compromises the efficacy of clopidogrel, leading to reduced formation of the active thiol metabolite and reduced inhibition of platelet aggregation [15]. While the impact of *CYP2C19* genotype on clopidogrel efficacy in lower risk populations who do not undergo PCI is questionable [25, 26], the data consistently show reduced clopidogrel effectiveness after PCI in carriers of a nonfunctional allele, with the lowest effectiveness observed in PMs [12, 16]. Based on these data, guidelines by the Clinical Pharmacogenetics Implementation Consortium (CPIC) recommend integrating available genotype results into prescribing decisions for antiplatelet therapy after PCI [14]. They specifically recommend prasugrel or ticagrelor for patients with a LOF allele in the absence of contraindications.

While CPIC guidelines do not provide recommendations on whether or not to order genotyping, leaving that to the discretion of the physician, this is addressed in practice guidelines for the management of patients undergoing PCI [1, 2]. These guidelines recommend against routine use of genetic testing for all patients undergoing PCI, citing an absence of data from large randomized controlled trials, but state that testing may be considered for patients at high risk for poor outcomes. Two multi-center trials assessing the efficacy of genotype-guided antiplatelet prescribing after PCI are ongoing but are not expected to be completed for at least a year (ClinicalTrials.gov Identifiers: NCT01742117 and NCT01761786). There are, however, recent data on outcomes from a pragmatic clinical trial showing a reduced risk of the composite of ischemic and bleeding events with genotype-guided antiplatelet therapy versus usual care in patients with ACS, most of whom underwent PCI [27]. There are additional data from pragmatic studies of *CYP2C19* genotyping in clinical care. Specifically, we showed in a study of 412 patients who underwent *CYP2C19* genotyping and PCI at UF Health in Gainesville that there was a higher incidence of major adverse cardiovascular events (MACE), consisting of cardiovascular death, myocardial infarction, stroke, or stent thrombosis, in patients with a nonfunctional allele treated with clopidogrel versus prasugrel or ticagrelor [28]. In a subsequent study of 1815 patients genotyped across seven medical centers, we observed an increased risk for MACE, defined as death from any cause, non-fatal myocardial infarction, or non-fatal stroke, in nonfunctional allele carriers treated with clopidogrel versus alternative therapy (adjusted hazard ratio of 2.26, 95% confidence interval 1.18–4.32) [29]. There remained significantly worse outcomes with clopidogrel compared to alternative therapy when limiting the analysis to IMs. This is noteworthy because the FDA-approved clopidogrel labeling includes a boxed warning about reduced drug effectiveness in PMs, with two nonfunctional alleles, but does not address IMs, with a single nonfunctional allele [30]. A cost-effectiveness analysis based on event rates in this study is underway. Previous cost-effectiveness analyses using event rate probabilities derived from clinical trials or cohort studies showed that a genotype-guided approach to antiplatelet therapy after ACS and PCI is cost effective compared to universal clopidogrel, ticagrelor, or prasugrel [31, 32].

There were a number of valuable lessons learned based on our experience implementing *CYP2C19* testing at two different hospitals within UF Health. First, a number of conditions must be met for rapid genotyping to be feasible in the clinical setting. A genetic test is considered a high complexity test according to the Clinical Laboratory Improvement Amendments (CLIA) and must be performed according to a college of American pathologist/CLIA accredited process in the US [33]. Thus, while rapid genotyping platforms may be designed as point-of-care tests outside the US, they cannot be used in this manner in the US in the absence of a licensed molecular medical technologist available to perform the test. This generally requires the platform to be placed in the CAP/CLIA certified pathology laboratory, as done at UF Health Jacksonville. Relative to the Spartan RX™ system, the only FDA-cleared platform for rapid *CYP2C19* genotyping, the genetic sample must be placed in the instrument within an hour of collection, necessitating dedicated staff to efficiently deliver the sample to the pathology laboratory. While this process works well at UF Health Jacksonville given the close proximity of the cardiac catheterization and clinical pathology laboratories, it is not feasible at UF Health Shands Gainesville where the pathology laboratory is located farther away. Another limitation of the Spartan RX™ system is that only a single sample can be genotyped at a time. Thus, to increase genotyping capacity at the Jacksonville site, a second system was added. However, this still limited the number of patients we could genotype and contributed to our inability to offer genotyping to all patients undergoing left heart catheterization. Finally, 13.9% of samples initially failed to genotype with the Spartan RX™ system, requiring repeat sample collection and increasing the cost of testing.

The approach taken at UF Health Jacksonville was to genotype all patients undergoing left heart catheterization with intent for PCI. While 58% of patients did not immediately proceed to PCI, in the event that patients return for PCI in the future, genotype will be readily available in the EHR to help inform therapy. The availability of a rapid genotyping platform, however, negates the need to genotype all patients at the time of cardiac catheterization and allows for efficient genotyping at the time of PCI.

Another important lesson is that when genotype results are not available for several days after PCI, as was the case at UF Health Gainesville, it creates significant disruptions in workflow caused by the need to adjust therapy after the patient has left the cardiac catheterization laboratory and potentially after the patient has been discharged from the hospital. In fact, this was the primary barrier cited by two large private practices in Florida with high volume cardiac catheterization laboratories, who we previously approached about implementing *CYP2C19* testing. This suggests that to implement testing more broadly, genotyping should cause minimal to no negative impact on work flow. Solutions to this problem are either preemptive pharmacogenetic testing, so that results are available ahead of PCI, or rapid genotyping at the time of PCI as done at UF Health Jacksonville. In the landscape of a lack of reimbursement for preemptive pharmacogenetic testing, the use of rapid genotyping platforms may be the most feasible solution at this time.

A third lesson learned was that despite the shift to newer agents early after ACS and PCI, genotype still has an important role in informing on the choice of long-term antiplatelet therapy and specifically with informing de-escalation from more potent antiplatelet therapy early after PCI to clopidogrel for maintenance therapy. This has important prognostic implications given that most ischemic events occur early (i.e., first month), while bleeding events continue to accrue over time particularly with the long-term use of prasugrel and ticagrelor [34, 35]. These observations have led to experts to advocate that the use of prasugrel or ticagrelor be limited to the first few weeks or month after PCI to reduce the risk of ischemic events, and then therapy de-escalated to clopidogrel to minimize the risk of bleeding complications [3, 36]. However, a non-guided de-escalation approach (e.g. without genotyping or platelet function testing) to a less potent agent has been associated with conflicting outcomes findings [37, 38], with very early de-escalation after an ACS event associated with an increased risk of ischemic events. On the contrary, the recently reported testing responsiveness to platelet inhibition on chronic antiplatelet treatment for acute coronary syndromes (TROPICAL-ACS) trial, using a platelet function guided approach, provides a justification for a de-escalation strategy [39]. The trial specifically demonstrated that de-escalation from prasugrel to clopidogrel, guided by platelet function testing, was non-inferior to continued prasugrel use in preventing adverse cardiovascular events after ACS and PCI. A limitation with platelet function testing is that it must be performed while the patient is on treatment. That is, patients in the TROPICAL-ACS trial were switched to clopidogrel for 1 week prior to testing, and only those with sufficient evidence of platelet inhibition were continued on clopidogrel, whereas those with high on-treatment platelet reactivity were switched back to prasugrel. A strategy of de-escalation and escalation back to prasugrel or ticagrelor with repeated measures of platelet function may not always be practical and can be inconvenient for the patient [40]. Genotyping may represent another means of guiding de-escalation, and unlike platelet function testing, it can be done *apriori*, without the need to switch the patient to clopidogrel prior to testing.

In the current study, we observed continued high use of prasugrel or ticagrelor in CYP2C19 PMs, who have significantly limited capacity to activate clopidogrel. However, in line with a genotype-guided de-escalation approach, use of alternative antiplatelet therapy declined significantly over the 6 month period after PCI among those without a nonfunctional allele. Interestingly, there was a similar decline in IMs. The preference for continuing alternative therapy in PMs, but not necessarily in IMs, could be based on the boxed warning on the FDA-approved clopidogrel labeling, which is specific to PMs [30]. Data from the pragmatic study described above showing improved outcomes in IMs treatment with alternative therapy versus clopidogrel support continuation of alternative therapy in IMs [29]. However, these data were not available during the first year of implementation at UF Health Jacksonville. Of note, a limitation of our findings is that we did not specifically assess whether factors, other than genotype, contributed to decisions to switch antiplatelet therapy, such as patient ability to afford alternative agents and presence of conditions that increase bleeding risk (e.g., use of concomitant oral anticoagulants, frailty, and history of intracranial or other serious bleeding), nor did we collect data on loading doses or on patients not undergoing genotyping for comparison.

An additional lesson learned was the importance of clinical pharmacy support and electronic clinical decision support when there are delays in obtaining genotype results to assist with communicating results to providers after the patient has left the cardiac catheterization laboratory and in some cases, after the patients has been discharged from the hospital. In settings where rapid genotyping is available and initial use of alternative therapy post PCI is high, one could argue that pharmacist support may not be necessary to assist with early antiplatelet treatment decisions. Nonetheless, approximately 40% of IMs in the current study were placed on clopidogrel acutely, which is inconsistent with CPIC guidelines, suggesting that clinical pharmacist support and/or electronic clinical decision support may be warranted regardless of genotype turnaround time. Pharmacist support and electronic decision support may also be important in the chronic management of patients to assist genotype-guided decisions to de-escalate antiplatelet therapy to clopidogrel. Indeed, at UF Health, Gainesville, where there is significant pharmacist support plus electronic decision support, the majority of IMs were switched from clopidogrel to prasugrel or ticagrelor, consistent with CPIC recommendations. However, the opposite occurred in the absence of such support at UF Health, Jacksonville. Pharmacists can also play important roles in educating providers and patients on genetic test results and implications for treatment response regardless of genetic testing procedures.

## Conclusion

Our data show that providing genotype-guided antiplatelet therapy after PCI with the use of a rapid genotyping platform is feasible with high provider acceptance of genetic testing. This is among the first reports of implementation metrics with clinical use of a rapid *CYP2C19* genotyping platform and the largest study of rapid *CYP2C19* genotyping in clinical care to date. With increasing use of newer antiplatelet agents, especially in the setting of ACS, our data suggest that genotyping may have an important role in informing maintenance antiplatelet therapy following the early post-PCI period when risk for adverse events is highest.
